# A case of isocyanate-induced asthma possibly complicated by food allergy after peanut consumption: a case report

**DOI:** 10.1186/1745-6673-3-29

**Published:** 2008-11-26

**Authors:** Ervin Ç Mingomataj, Enkelejda Gjata, Fatmira Xhixha, Entela Hyso

**Affiliations:** 1 Dept. of Allergology & Clinical Immunology, "Mother Theresa" School of Medicine – Tirana, Albania; 2 Cabinet of Allergology, District's Policlinic – Lushnja, Albania; 3 Cabinet of Allergology, Policlinic of Specialties No 3, Tirana, Albania; 4 Cabinet of Allergology, Vlora Regional Hospital, Albania

## Abstract

**Background:**

Isocyanates are extensively used in the manufacture of polyurethane foams, plastics, coatings or adhesives. They are a major cause of occupational asthma in a proportion of exposed workers. Recent findings in animal models have demonstrated that isocyanate-induced asthma does not always represent an IgE-mediated sensitization, but still a mixed profile of CD4+ Th1 and TH2, as well as a CD8+ immune response. Despite immunologic similarities between this pathology and IgE-mediated food allergies, this co-morbidity is rarely reported.

**Case presentation:**

A 50-year old man employed as vehicle body painter, for 8 years complained about breathlessness, wheezing, sneezing, nasal obstruction and excessive production of mucus during the use of DuPont Refinish Centari Tintings – an acrylic enamel tint. Symptoms occurred 15–20 minutes after workplace exposure and usually persisted until evening, or at times, up to two consecutive days. The above mentioned symptoms were associated with a decrease of lung functions parameters. The use of inhaled adrenergic bronchio-dilatators and steroids relived the symptoms.

In addition, three years ago he developed an anaphylactic reaction due to peanut consumption, experiencing urticaria, angioedema and airway obstruction. He was successfully treated in the hospital. Later, the subject exhibited labial itching, as well as orbital and perioral angioedema, 20 minutes after stationary performance of challenge test with peanuts.

Evaluating the reported data, this process might be developed rather due to induction of a TH2 profile, because in both cases have occurred IgE-mediated symptoms. A less plausible mechanism could be the presence of isocyanates in peanuts due to a probable contamination by pesticides resulting in an allergic reaction after "consumption" of di-isocyanate as long as the isocyanate contamination of peanuts has not been proven.

**Conclusion:**

Despite the lack of relevant laboratory findings, this might be the first case of isocyanate-induced occupational asthma described in a patient who developed peanut allergy symptoms later in his life. However, in order to take further suitable precautions, further studies are necessary to elucidate the questions posed in this report.

## Background

Isocyanates, widely used in the manufacture of polyurethane foams, plastics, coatings, or adhesives, and are known to cause the most common type of occupational asthma in a number of exposed workers [1-4]. Additionally, some residents living nearby fiber processing and polyurethane foam manufacturing facilities become sensitized to toluene di-isiocyanate (TDI) [5].

With respect to pathogenesis, recent findings especially in animal models demonstrated that isocyanate-induced asthma does not always represent an IgE-mediated sensitization, but still a mixed profile of CD4+ Th1 and TH2, as well as CD8+ immune response [2,4,6-10]. Also a combined IL-4/IL-13 depletion in a murine model effectively prevented almost all asthma pathologic symptoms [4]. Furthermore, Herrick et al. demonstrated that eosinophilic inflammatory processes in the airways were mediated by TH2 cytokines and not by IFNγ [7,8].

This pathology has an important clinical relevance, because asthmatic symptoms are developed in 5–15% of exposed workers and these symptoms may persist even after complete isocyanate avoidance, perhaps due to metalloproteinase MMP-9 overproduction and consequent induction of airway inflammation and remodeling [9,11,12]. Apart from the inhalation route of exposure, many experimental findings in animals have demonstrated the potential role of dermal contact after isocyanate exposure regarding the initial response and subsequent development of occupational asthma, whereas the use of latex gloves did not prevent the isocyanate sensitization among exposed workers [9,13-16].

Despite some immunologic similarities between this pathology and IgE-mediated food allergies, this co-morbidity is rarely reported. In effect, peanut allergy occurs generally in childhood [17]. Because of these facts, the description of a case of isocyanate-induced occupational asthma in a patient who developed symptoms of peanut allergy later in his life could be of great interest.

## Case presentation

### Our case

A 50-year old man, a former smoker, employed as vehicle body painter 25 years ago, used to work with an acrylic enamel tint – DuPont Refinish Centari Tintings. 17 years later, he began to exhibit urticaria and facial angioedema after work exposure. These symptoms were resolved after treatment with antihistamines. The last 8 years he experienced also cough, breathlessness, wheezing, sneezing, nasal obstruction and excessive production of mucus. The symptoms occurred 15–20 minutes after exposure to acrylic enamel tint at the workplace and persisted usually until the evening, or at times, up to two consecutive days. The use of inhaled adrenergic bronchio-dilatators and steroids relived the symptoms within two hours. In fact, he irregularly used to inhale budesonide in case of clinical deteriorations. He had used an oronasal mask to avoid tint for four years. The subject experienced different respiratory obstructive symptoms such as nasal obstruction and excessive production of mucus, sneezing, as well as dispnoea and chest tightness, if he discontinued the using of the face mask at workplace.

Spirometry showed normal ventilatory function in the non-working days. A significant reduction in forced expiratory volume at first second (FEV1) (50%), functional vital capacity (FVC) (67%) and peak expiratory flow (PEF) (75%) was observed after work exposure. The above mentioned respiratory functional parameters were normalized after the use of inhalant bronchio-dilatators and steroids. Consequently, within two hours the symptoms were resolved. Skin prick tests with common aeroallergens were negative. Blood test and radiological examinations were within normal limits. There was no familial history of atopy.

In addition, three years ago he developed an anaphylactic reaction due to peanut consumption. The patient manifested classical symptoms of anaphylaxis as urticaria, angioedema and dispnoea. He showed a marked clinical improvement only after four hours of treatment in hospital. Some months later, the patient underwent the oral challenge test with peanuts and 20 minutes later, he developed labial itching, as well as orbital and perioral angioedema. Afterwards, he followed a strict peanut elimination diet. All these findings confirm the diagnoses of occupational asthma and rhinitis due to isocyanates and peanut anaphylactic reaction.

## Discussion

TDI and diphenyl-methane di-isocyanate (DMI), used as drying accelerator of Centari tint, are widely known as respiratory irritators [1-4]. Isocyanates are a group of aromatic and aliphatic compounds of low molecules weight containing the group -N = C = O [9]. The ability to react with acrylic polyols and therefore to form the coating makes this compound a drying accelerator for paints and varnishes [1]. On the other hand, many reports point out that high concentrations of these compounds in the air, especially of TDI, irritate and sensitize the airways of exposed workers [4,9,11,12,16].

The most plausible immune mechanism involved in the sensitization to di-isocyanates is the TH2 profile induction, but simultaneous induction of TH1 and CD8 responses are also reported [2,4,6,7,9,15]. Recent studies in animal models have shown that sensitization can occur through subchronic inhalation of di-isocyanate at levels as low as 20 ppb as well as through dermal exposure [2,7,10,14-16,18]. Unfortunately, in Albania there is no reliable authority to control the environmental conditions of workplaces, especially in small private establishments. In this respect, rigorous preventive measures are needed [1,3].

The association of isocyanate-induced occupational asthma and anaphylaxis to peanuts consumption has not been described previously. Such complication of isocyanate-induced occupational asthma by food allergy is reported at least in two cases and the implicated foods were the plants of the mustard family [19,20]. The suggested phys-pathological mechanism of these cases was the cross-reactivity, as isothiocyanates are found in mustard spice [19]. As long as such compounds are not seen in peanuts, the induction of anaphylaxis due to peanut consumption as a consequence of prior isocyanate-induced occupational sensitization was not demonstrated.

The association of both pathologies could be explained with induction of TH2 immune response rather than with induction of TH1 one. This is supported by typical clinical findings such as the occurrence of IgE-mediated symptoms following peanut consumption in a subject previously diagnosed with an isocyanate-induced asthma, even if in our case there was no laboratory evidence (because of objective reasons) [2,4,6-9].

On the other hand, a less plausible mechanism could be the presence of isocyanates in peanuts due to a probable contamination by pesticides and therefore, an allergic reaction was induced after the di-isocyanates "consumption". In this respect, isocyanates such as methylisocyanate, used as intermediates in the synthesis of carbamate pesticides or di-isocyanates, are highly reactive compounds that spontaneously bind to biological macromolecules [9,21]. Because the isocyanate peanuts contamination has not been proven, very limited data support this hypothesis.

## Conclusion

The lack of relevant laboratory findings could be a limitation for this report. Nevertheless, this case description could be of interest, because it is possibly the first reported case of isocyanate-induced occupational asthma in a patient who developed symptoms of peanut allergy later in his life. However, further detailed studies are necessary to elucidate the questions posed in this report, in order to take further suitable precautions.

## Abbreviations

CD: cluster of differention; DMI: diphenyl-methane di-isocyanate; FEV1: forced expiratory flow at first second; FVC: functional vital capacity; Ig: immunoglobulin; IFN: interferon; IL: interleukin; MMP: metalloproteinase; PEF: peak expiratory flow; TDI: toluene di-isiocyanate; TH: lymphocytes T helper.

## Competing interests

The authors declare that they have no competing interests.

## Authors' contributions

EÇM has been involved in drafting and revising the manuscript, EG has carried out and analyzed patient's data. FX and EH have contributed in the analysis and interpretation of acquired data.
